# The underappreciated role for phase variation and quasi-clonality in microbial populations

**DOI:** 10.1371/journal.ppat.1014761

**Published:** 2026-11-06

**Authors:** Sophie Dyzenhaus, Scott D. Gray-Owen, Freeman Lan

**Affiliations:** 1 Department of Molecular Genetics, Temerty Faculty of Medicine, University of Toronto, Toronto, Ontario, Canada; 2 Institute of Biomedical Engineering at Faculty of Applied Science and Engineering, University of Toronto, Toronto, Ontario, Canada; Duke University School of Medicine, UNITED STATES OF AMERICA

## Abstract

Phase variation is a well-established phenomenon in some bacteria, where it generates heritable phenotypic variants within a population. These states, while reversible, are stable across generations, giving rise to quasi-clonal populations even when starting from a single bacterium. Underlying mechanisms include variation in short homopolymeric or heteropolymeric sequence repeats, DNA inversions mediated by site-specific recombination, reversible insertions and deletions, and switching between alternative methylation states. Although phase variation occurs in diverse bacteria, its contribution to phenotypic heterogeneity remains overlooked and use of the term is often absent in describing this phenomenon.

Microbiologists who work with bacteria that are known to undergo phase variation (PV) must routinely account for how this phenomenon might contribute to phenotypes observed and how it might explain apparent differences between strains or even between experiments with the same isolate [1]. As a prototypical example, *Neisseria gonorrhoeae* is a human-specific pathogen with a small genome and few transcription factors to specifically regulate pathogenesis. Rather than simply responding to environmental stimuli, the gonococci maintain a phenotypically diverse population that allows selection for the most effective combination of metabolic and virulence factors being expressed or not. With as many as 85 genes being seen to phase vary [2], individual variants within the population may differ in their ability to persist within any environment due to variance in expression of these factors. These variants can affect host attachment, surface structures, nutrient acquisition, natural competence or regulatory restriction-modification systems, the latter of which can lead to broad downstream changes through epigenetic regulation. Thus, PV may allow selection for the expression of useful capacities for a given niche or against expression of antigens that are targeted by the adaptive immune response. Compared to gene regulation, phase variation encodes enormous variation with relatively little investment (no transcriptional regulators, no gene circuits) and continues in the absence of environmental cues. This may be an advantageous method for increasing adaptive capacities while maintaining small genomic and proteomic footprints.

While phase variable loci have long been associated with virulence in the pathogenic *Neisseria sp.* and *Haemophilus influenzae* [1], a greater appreciation for the effect of phase variation is beginning to emerge widely in the microbiology community. Highlighting this are reviews focused on the potential importance of PV in *Staphylococcus aureus* [3] and *Clostridium difficile* [4], two important Gram-positive pathogens where it has not often been considered. However, the connection between this well-established method of regulation and newly discovered phase-variable loci is not always indicated. For example, recent papers reveal fascinating new examples of PV in *Streptococcus pneumoniae* [3,5], *H. influenzae* [6] and the human gut microbiome [7]*,* but do not refer to the previously published concept of PV.

Here, we aim to highlight the importance of PV and the quasi-clonality that it generates across many species of bacteria as an introduction for the wider microbiology community. We hope that this will allow phase variation, both as a terminology and as an important mechanism of gene regulation, to become more broadly appreciated across the field.

## Defining phase variation (PV)

PV and related phenomena have been defined multiple times over the last century [1]. For present purposes, we define PV as specific, reversible genetic changes that occur at defined loci at rates exceeding those of spontaneous mutation and in the absence of selection. The major mechanisms of PV discovered thus far are slipped-strand mispairing, which alters simple sequence repeats (SSRs) during DNA replication [8], and site-specific recombination of inverted repeats to promote genomic inversions [9]. At promoters, PV can turn ON/OFF or increase and decrease gene expression, while within the coding region of a gene it can alter the reading frame for translation (Fig 1) [8,10]. Other recombination-driven events, such as site-specific recombination between two distant loci [11], or reversible transposon insertions [12], can produce similar outcomes. While this typically regulates a single protein, PV of methylases can induce epigenetic changes that impact expression of multiple genes simultaneously [4]. Each phase variable locus can exist in specific states; this differentiates them from hypermutator or mutational hotspots [13,14], which rely on random mutations and so do not consistently yield the same variants.

**Fig 1:**
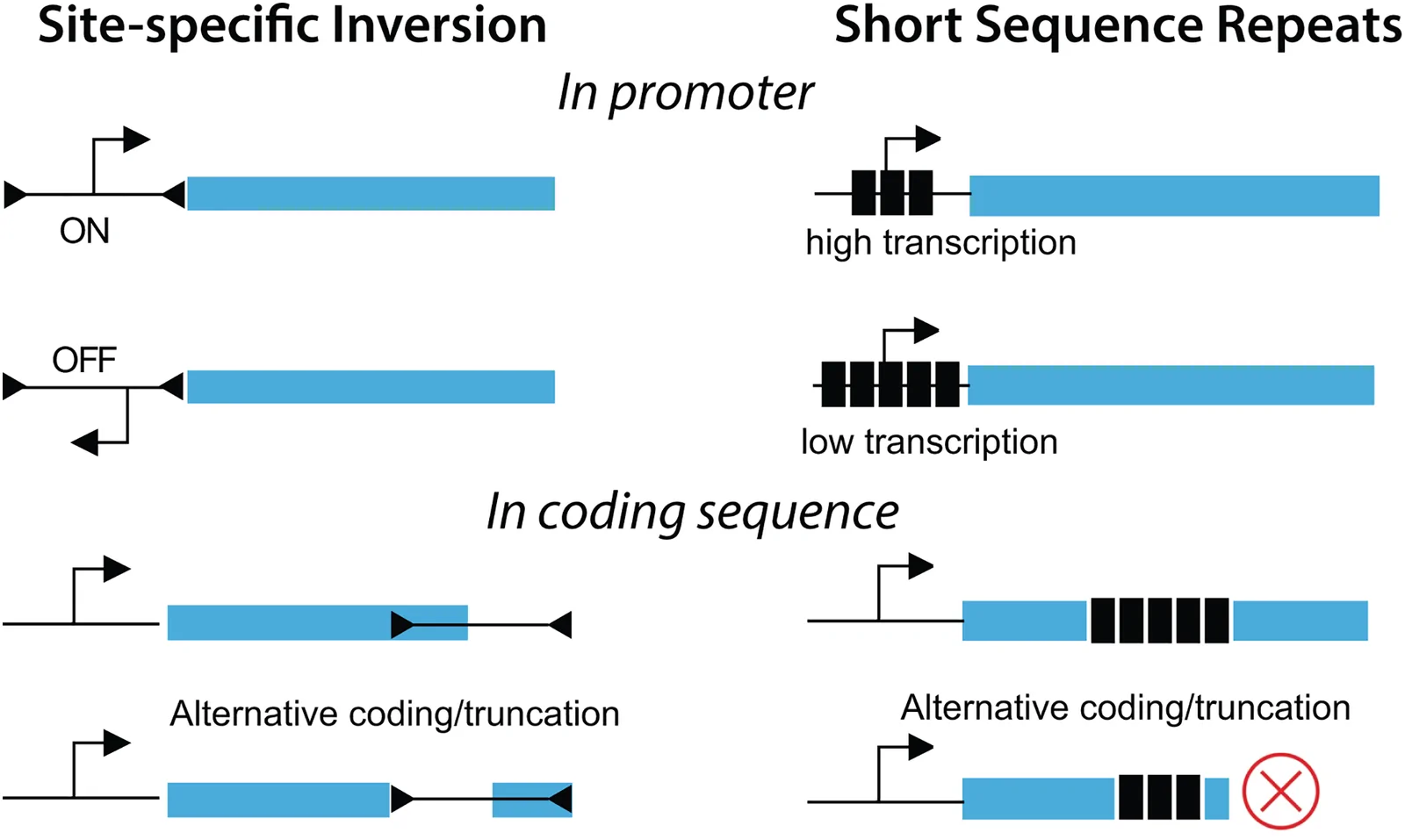
Major mechanisms of phase variation. Center-pointed triangles represent inverted repeats and black blocks represent short sequence repeats.

## Phase variation generates quasi-clonal populations

Ongoing PV gives rise to a quasi-clonal population comprised of different variants (Fig 2). These may arise from the phase-variable switching at a single locus or a combination of changes at multiple loci. Any particular combination of ‘on’ and ‘off’ states across a bacteria’s repertoire of PV loci is referred to as a phasotype [1]. While the variation at each locus is genetic and heritable, any single bacterium can produce the full spectrum of phasotypes and so will regenerate a quasi-clonal population structure after environmental selection and/or a physical bottleneck that severely reduces the population size. However, given that each variant may exhibit different fitness costs and benefits, selection acts so that particular variants will dominate the quasi-clonal population within each particular environment.

**Fig 2:**
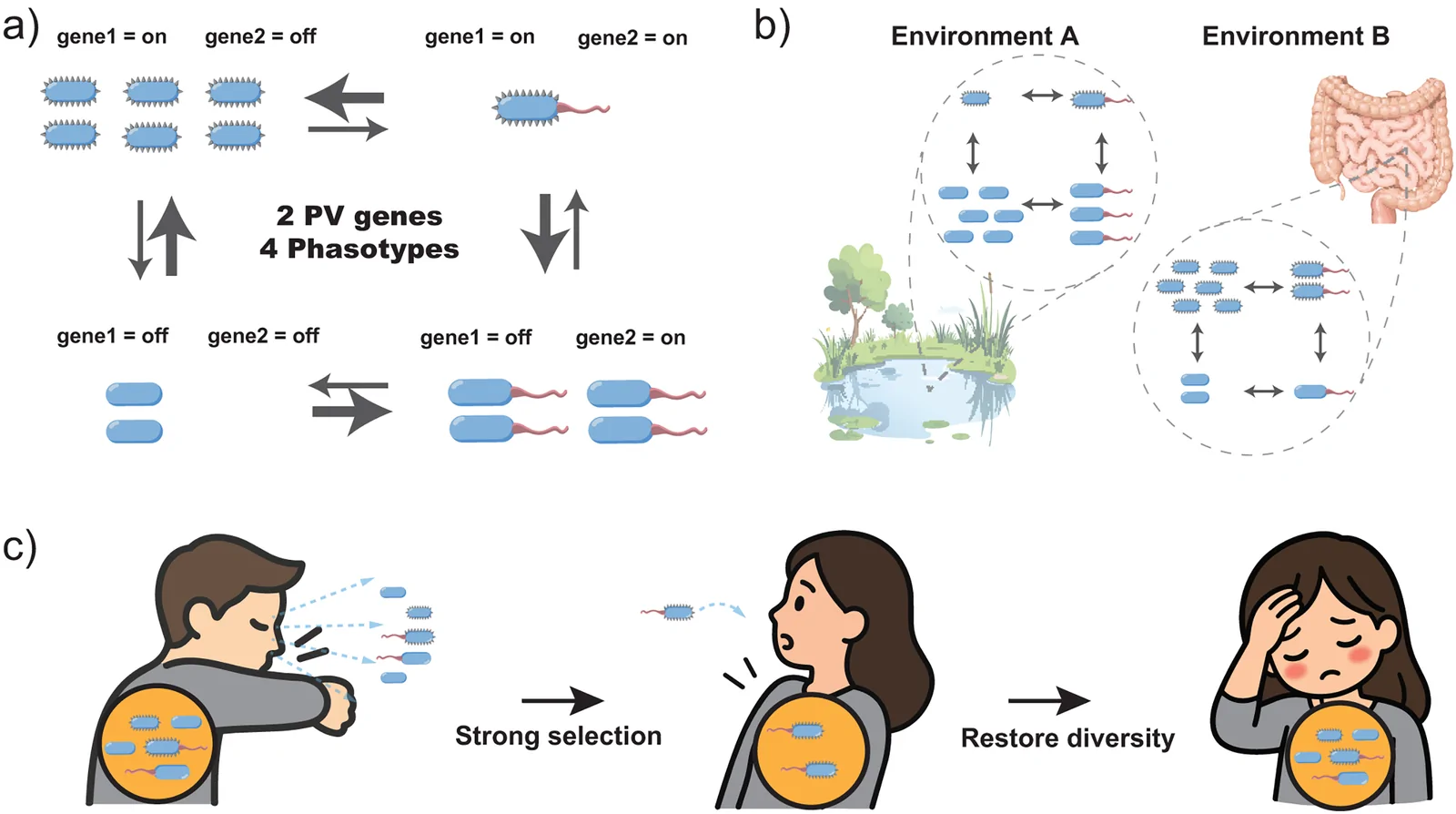
Phase variation gives rise to quasi-clonal populations. **(a)** Phase variation at multiple loci gives rise to distinct patterns of ‘on’ or ‘off’ expression, known as phasotypes. Switching rates at each locus will bias the composition of the quasi-clonal population. **(b)** Different environments will favor different phasotypes, with these selective events strongly influencing the population composition. **(c)** Strong selective pressures and/or environmental bottlenecks can temporarily reduce the quasi-clonal population diversity, but any bacterial cell can re-establish the full spectrum of phasotypes over time.

A clear demonstration of this phenomenon is apparent with the human gut commensal *Bacteroides fragilis*, where a recombinase-dependent inversion of promoter sequences leads to the combinatorial phase variation of seven polysaccharide capsule synthesis operons, which generates a quasi-clonal population consisting of 128 distinct phasotypes. Single-cell sequencing combined with computational modeling has shown that a single colony tends to be dominated by one phasotype, but constant *in vitro* broth conditions produce diverse quasi-clonal populations that maintain a composition of over 117 phasotypes at detectable frequencies, with the most dominant phasotype forming only 22% of the population after 14 days of *in vitro* growth [15]. This population composition is determined by the intrinsic rates of switching between each PV loci and the fitness of each phasotype generated.

In pathogens, one of the best-described examples of PV is the ON/OFF toggling of colony opacity-associated (Opa) proteins in *N. gonorrhoeae*. The Opa proteins are encoded by 11 distinct *opa* loci, each of which contains a pentanucleotide repeat sequence within their coding region. Each locus is constitutively transcribed, but the number of repeats present determines whether the downstream sequence is in or out of the translational reading frame. Since variation at each locus is independent, a single gonococci may express either zero, one or multiple Opa proteins [16]. Distinct from a binary ON/OFF switch, repeat sequences within bacterial promoters can also allow more nuanced changes to gene expression. For example, the *opc* gene of *N. gonorrhoeae* contains a poly-cytidine tract within its promoter, the length of which determines the spacing between consensus sequences that dictate transcriptional expression [16]. Since the number of repeats is susceptible to change with each cell division, such simple effects can maintain ongoing diversification of the population.

## Why have phase variation and quasi-clonality been overlooked?

PV escapes detection by standard genomic sequencing analysis where read-mapping algorithms typically generate consensus sequences that overlook minor variants within a population. Moreover, PV events frequently disrupt open reading frames, leading to mis-annotation or omission in automated genome annotation pipelines. While PV might be crucial under fluctuating selective pressures, it can be detrimental under strong, constant selective pressures such as common laboratory conditions where the most fit quasi-clone will ultimately prevail and then spontaneous mutations dominate as the primary adaptive mechanism [17,18]. These challenges, combined with the traditional view of bacterial strains as fully clonal populations, make PV more easily overlooked, particularly by researchers working in species where PV has not been well characterized. Indeed, overlooking the involvement of PV has led to misinterpretation of experimental results [19] and may explain different results among biological replicates with bacteria in which it is prevalent.

In the cases where PV is discovered and reported as a novel mechanism, it is apparent that the problem is an issue with communication. This reduces recognition of mechanistic parallels by those who work with bacteria where PV is well appreciated. Even in species where PV is appreciated, the sequence data processing pipelines frequently result in quasi-clonal populations being overlooked. New technologies, including DoTA-seq, where microfluidics technology allows for single-cell sequencing of bacteria, are beginning to demonstrate the vast extent of quasi-clonality, which should prevent minor variants being disregarding as sequencing errors [20].

## Conclusions

Despite a long history of research in certain pathogens, phase variation remains under-appreciated in microbiology at large. This mechanism of adaptation, whereby the environment selects for the fittest variant from a quasi-clonal population, may be much more ubiquitous among bacteria than currently understood. Indeed, given the diversity of bacteria that have already been described to display some form of PV, it is provocative to consider that quasi-clonal populations may be a fundamental aspect of bacterial biology. Moving forward, we believe there is a wealth of biology to be uncovered in phase variation of diverse bacterial species, revealing functions that are silent in selected isolates and/or helping to explain behaviors in bacterial intra-strain heterogeneity.
